# Complete ^1^H, ^15^N and ^13^C resonance assignments of *Bacillus cereus* metallo-β-lactamase and its complex with the inhibitor *R*-thiomandelic acid

**DOI:** 10.1007/s12104-013-9507-1

**Published:** 2013-07-10

**Authors:** Andreas Ioannis Karsisiotis, Christian Damblon, Gordon C. K. Roberts

**Affiliations:** 1 The Henry Wellcome Laboratories of Structural Biology, Department of Biochemistry, University of Leicester, Lancaster Road, PO Box 138, Leicester, LE1 9HN UK; 2 The School of Pharmacy and Pharmaceutical Sciences, Biomedical Sciences Research Institute, University of Ulster, Coleraine, BT52 1SA Northern Ireland UK; 3 Chimie Biologique Structurale, Institut de Chimie, Sart-Tilman (B6c), Université de Liège, 4000 Liège, Belgium

**Keywords:** Metallo-β-lactamase, *R*-thiomandelic acid, BcII, BcII–inhibitor complex

## Abstract

β-Lactamases inactivate β-lactam antibiotics by hydrolysis of their endocyclic β-lactam bond and are a major cause of antibiotic resistance in pathogenic bacteria. The zinc dependent metallo-β-lactamase enzymes are of particular concern since they are located on highly transmissible plasmids and have a broad spectrum of activity against almost all β-lactam antibiotics. We present here essentially complete (>96 %) backbone and sidechain sequence-specific NMR resonance assignments for the *Bacillus cereus* subclass B1 metallo-β-lactamase, BcII, and for its complex with *R*-thiomandelic acid, a broad spectrum inhibitor of metallo-β-lactamases. These assignments have been used as the basis for determination of the solution structures of the enzyme and its inhibitor complex and can also be used in a rapid screen for other metallo-β-lactamase inhibitors.

## Biological context

The β-lactamases, enzymes which inactivate β-lactam antibiotics by hydrolysis of their endocyclic β-lactam bond, are a major cause of resistance to these antibiotics in pathogenic bacteria (Frère et al. 1999). The rapid increase in this resistance is a major clinical and public health concern, as these antibiotics have long been crucial in the treatment of serious bacterial infections. The zinc dependent metallo-β-lactamases (MBLs) (Bebrone 2007) are of particular concern since many of them are located on highly transmissible plasmids and they have a broad spectrum of activity against almost all β-lactam antibiotics including carbapenems. Resistance due to MBLs is severely limiting treatment options in Asia, Europe, and Latin America (Fritsche et al. 2005).

*R*-thiomandelic acid has been identified (Mollard et al. 2001; Damblon et al. 2003) as a potent and promising MBL inhibitor with a K_i_ of 0.09 μM towards the MBL from *Bacillus cereus,* BcII, a representative of the subclass B1 of MBLs which includes the clinically important enzymes of the IMP and VIM families and the recently identified NDM-1 enzyme. BcII has been well studied crystallographically (Carfi et al. 1995, 1998; Fabiane et al. 1998) but its solution structure has not hitherto been determined. The complete backbone and sidechain resonance assignments for the BcII MBL (227 amino-acid residues) and its complex with *R*-thiomandelate are presented in this communication. The solution structures of BcII and of the BcII–thiomandelate complex, the product of these assignments, are reported elsewhere (Karsisiotis, A.I., Damblon, C.F. and Roberts, G.C.K., submitted for publication). The only other example of sequence specific assignments for an MBL is of the CcrA enzyme from *Bacteroides fragilis* (Scrofani et al. 1998) (BMRB 4102) where the majority of HN, N, CA, CB, CO and HA resonances were assigned.

## Methods and experiments

### BcII expression, labeling and purification

^15^N-^13^C-labeled protein was expressed from plasmid pET9a/BCII (Mollard et al. 2001) in *E. coli* BL21 (DE3) competent cells. The bacterial cells were grown at 37 °C in autoclaved M9 (1 g/l NaCl, 14.6 g/l anhydrous Na_2_HPO_4_, 5.5 g/l anhydrous KH_2_PO_4_, pH 7.2) minimal medium, supplemented with ^13^C-D-Glucose (4 g/l) and ^15^NH_4_Cl (1 g/l) as the sole carbon and nitrogen source respectively and with 0.24 g/l MgSO_4_·7H_2_O and 0.02 g/l CaCl_2_·2H_2_O. Following induction (0.8 mM IPTG added at OD_600_ = 1.25), the cells were grown for a further 4 h, harvested and broken by 4 passes through a French pressure cell. BcII was purified to over 95 % purity (as shown by SDS-PAGE analysis and 2D ^1^H-^15^N HSQC spectra), using an SP-HP Sepharose column (Amersham Pharmacia Biotech, Uppsala, Sweden) pre-equilibrated in 20 mM MES [2-(*N*-morpholino)ethanesulfonic acid] buffer, pH 6.4, containing 0.2 mM ZnCl_2_. The protein was eluted by a linear NaCl gradient (0–1 M) in 20 mM MES, 0.2 mM ZnCl_2_, pH 6.4; the active fractions of the main peak were concentrated and buffer exchanged to 20 mM MES, 0.2 mM ZnCl2, pH 6.4 using a Vivaspin 5000 cutoff concentrator (2,850 rpm) and loaded for a second run to the same column pre-equilibrated in identical fashion. The protein was eluted by a shallower linear NaCl gradient (0–1 M) in 20 mM MES, 0.2 mM ZnCl2, pH 6.4. Again the pooled active fractions were concentrated and buffer exchanged, using a Vivaspin 5000 cutoff concentrator, into NMR buffer (20 mM MES, 100 mM NaCl, 0.2 mM ZnCl_2_, pH 6.4).

### NMR spectroscopy

NMR experiments were recorded on Bruker Avance DRX 600 and 800 MHz instruments equipped with cryoprobes and a Bruker Avance DRX 600 MHz spectrometer. In addition a ^13^C-edited NOESY spectrum of the free enzyme was acquired on a Varian INOVA 800 MHz instrument (Biomedical NMR Centre, NIMR, London). All experiments were performed at 308 K using samples with an enzyme concentration of ~1 mM in 20 mM MES, pH 6.4, containing 10 % ^2^H_2_O, 100 mM NaCl, 0.2 mM ZnCl_2_. *R*-thiomandelate and samples of the BcII–*R*-thiomandelate complex were prepared as described previously (Mollard et al. 2001; Damblon et al. 2003). The 3D triple resonance experiments (Sattler et al. 1999) used for the sequential backbone assignments of BcII were the following pairs: HNCACB/CBCACONH, HNCA/HN(CO)CA and HNCO/HN(CA)CO. The HBHA(CO)NH/HBHANH pair of triple resonance experiments was used for the CαH and CβH assignments of BcII. The same set of experiments, with the exception of the HN(CO)CA and HBHANH experiments, was used for obtaining the assignments of the BcII–thiomandelate complex. Sidechain assignments for both BcII and the BcII–thiomandelate complex were obtained using (H)CCH-TOCSY and H(C)CH-TOCSY experiments with a TOCSY mixing time of 60 ms. A ^13^C-edited NOESY spectrum optimized for the aromatic region and a 2D [^1^H-^13^C] CT-TROSY-HSQC experiment were used for obtaining sidechain assignments of the aromatic residues. NOEs were assigned in ^15^N-edited and ^13^C-edited NOESY spectra with a mixing time of 80 ms. The total duration of each of the triple resonance experiments and of the NOESY experiments was between 48 and 96 h. ^1^H NMR and [^1^H-^15^N] HSQC experiments were performed as described previously (Damblon et al. 1999; 2003). All spectra were processed using the NMRPipe/NMRDraw software package (Delaglio et al. 1995), with processing scripts on a Linux workstation, or using Topspin (Bruker BioSpin). Typically a sine-bell or a sine-squared bell window function was applied to the time domain data to improve resolution. Zero filling was usually applied in all dimensions to improve digital resolution. Linear prediction was only applied when it improved resolution in the ^13^C dimension for the triple resonance experiments; it was not used for NOESY experiments. All assignments were obtained using the graphical NMR assignment program Sparky (Goddard and Kneller 2008).

### Assignment strategy and extent of resonance assignments

**Fig. 1:**
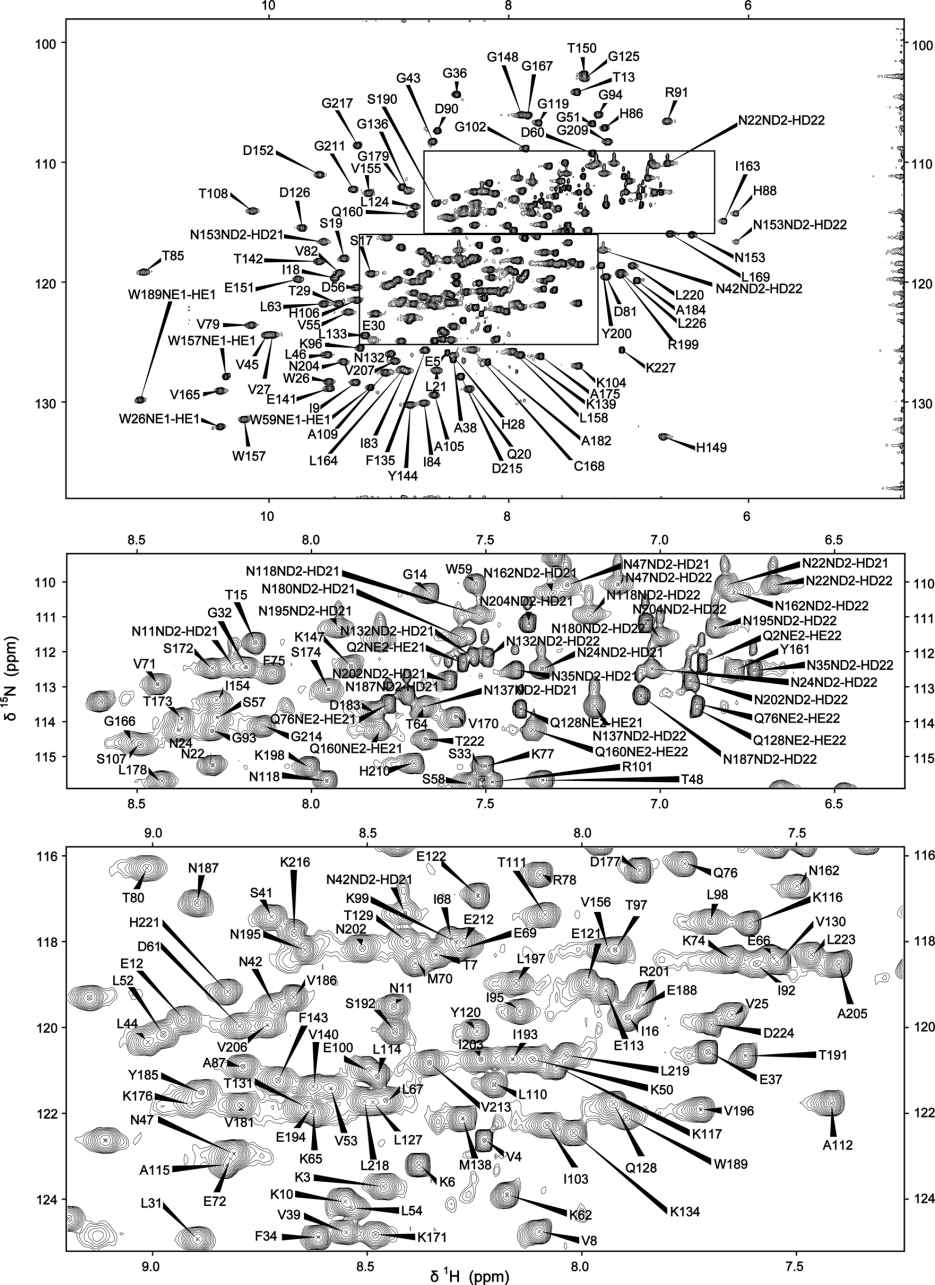
^1^H-^15^N HSQC spectra of the BcII metallo-β-lactamase. The full spectrum is shown at the *top*, with crowded regions shown as expansions below. The resonance assignments of the backbone, side chain amide and tryptophan indole NH resonances are indicated

**Fig. 2:**
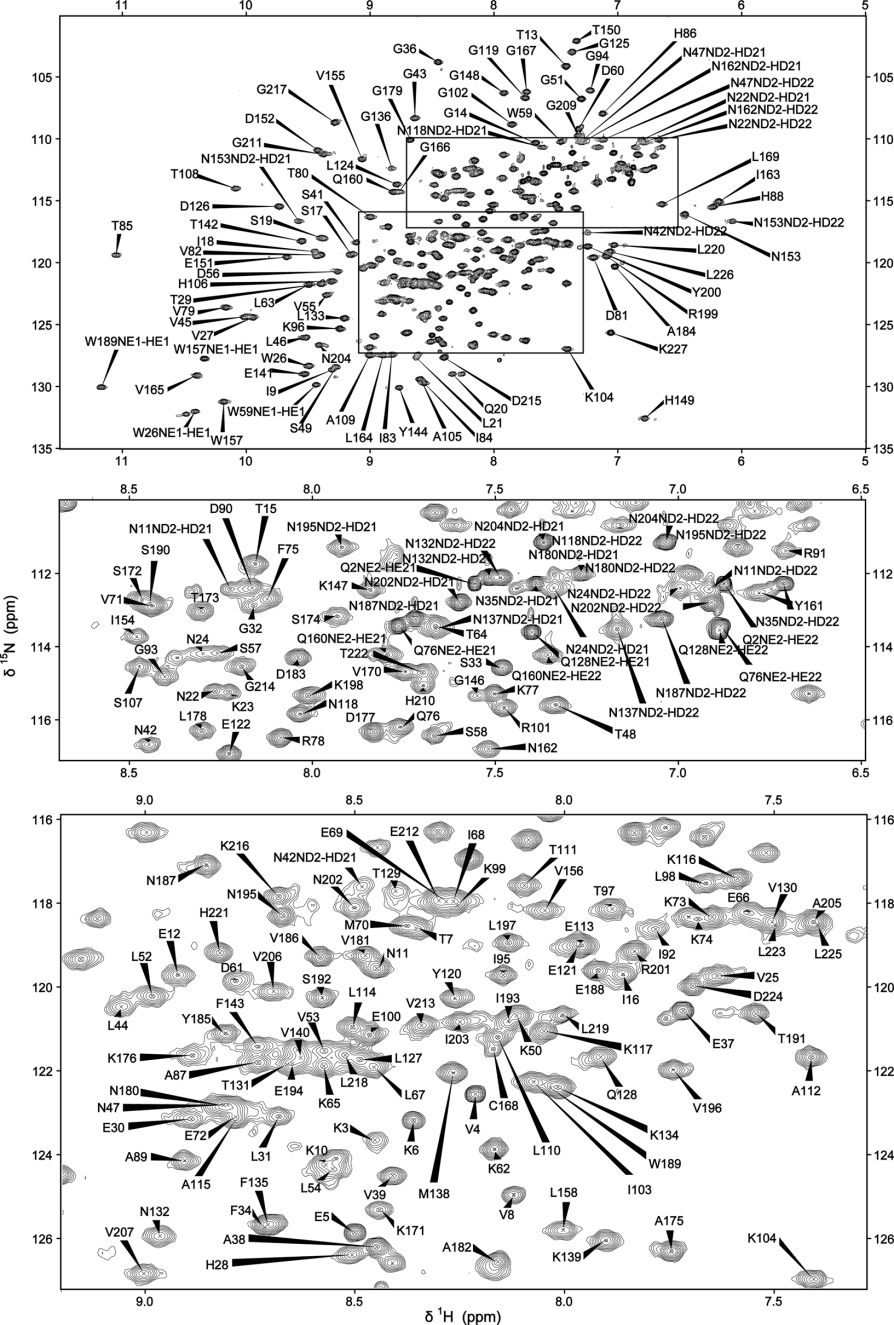
^1^H-^15^N HSQC spectra of the complex of BcII metallo-β-lactamase with *R*-thiomandelate. The full spectrum is shown at the *top*, with crowded regions shown as expansions below. The resonance assignments of the backbone, side chain amide and tryptophan indole NH resonances are indicated

The backbone and sidechain resonances of BcII and of the BcII–R-TM complex have been sequentially assigned using the suite of triple resonance experiments listed above (Figs. 1, 2). The analysis of the NOESY spectra proved to be very important in the maximum completion of the resonance assignments; at a later stage this served to verify existing assignments and further reduce the percentage of the unassigned resonances. Methionine ε-CH_3_ groups were assigned based solely on analysis of NOEs. The degree of completion of assignments for both BcII and the BcII–thiomandelate complex exceeds 96 % of all protons and 98 % of all carbons. For non-labile protons and for carbons, the majority of the missing assignments involve residues of the unstructured N-terminus (S1, Q2 and K3) or side-chains which are partly or wholly unstructured and are surface exposed and located on loops and turns of the protein (e.g. K23, K73 and K99). Only 9 backbone amide proton assignments are missing for the free BcII (residues S1, Q2, K23, N35, S49, A89, N137, G146 and N180). This could be due to fast relaxation properties for these residues, the majority of which are located in turns or the loops of the protein. The sidechain resonances for these residues are usually observed with the exception of the two N-terminal residues and the surface-exposed K23. For the complexed BcII the same backbone NH assignments are missing with the exception of the resonances for A89 and N180 which appear upon inhibitor binding. Since the backbone resonances of G146 are unassigned, P145 cannot be reached by sequential connectivities and therefore the signals corresponding to this residue cannot be assigned conventionally. However, P145 chemical shifts were subsequently assigned when the side chain assignments for the remaining four proline residues were completed by assigning as a starting point the characteristic CD and HD resonances (~50 and ~3.5 ppm). All proline assignments were checked through analysis of NOEs. Homonuclear [^1^H-^1^H] NOESY and TOCSY experiments performed on deuterated samples were not sufficient to resolve the aromatic spin systems and obtain assignments for the 20 aromatic residues. Two experiments selective for aromatic residues were used: a 3D [^1^H-^13^C] NOESY-HSQC and a 2D [^1^H-^13^C] CT-TROSY-HSQC. The assignment of the histidine HD2 and HE1 protons was accomplished using information from a [^1^H-^15^N] HSQC experiment optimized for the detection of imidazole protons, the 2D [^1^H-^13^C] CT-TROSY-HSQC experiment and past work on BcII (Damblon et al. 1999; 2003). The assignments were verified with the use of NOESY data. The tryptophan side-chain resonance assignments were based on the characteristic CZ2 (~113 ppm) chemical shifts as a starting point and NOE interactions between neighbouring protons to identify all the proton shifts. Carbon shifts were obtained from the 2D [^1^H-^13^C] CT-TROSY experiment, and NOE correlations with the beta protons were observed for the aromatic HE3 and HD1 protons in aromatic ^13^C-edited NOESY spectra. The indole NH (HE1 proton) resonances were obtained from a basic HSQC experiment and NOEs to the neighbouring HD1 and HZ2 protons were observed. Essentially complete aromatic assignments were obtained (with exception of F34 HZ and Y200 QE). These assignments were additionally verified by a 3D [^1^H-^13^C] NOESY-HSQC optimized for the aromatic region.

The essentially complete NMR sequence specific assignments reported here were used to determine the solution structures of BcII and its complex with *R*-thiomandelic acid, and these structures throw light on the origins of the chemical shift changes on inhibitor binding (Karsisiotis, A.I., Damblon, C.F., and Roberts, G.C.K., submitted for publication). The assignments have also been used to study the binding of other inhibitors and compounds to BcII (Poeylaut-Palena et al. 2007; Liénard et al. 2008).

### Data bank deposition

The chemical shift assignments have been deposited as two different data sets for each of the two systems (BcII and BcII–thiomandelate complex), one derived from ^13^C-edited NOESY data and one derived from ^15^N-edited NOESY data, since intraresidual, *i* + 1 and *i* − 1 assignments directly in the NOESY spectra were used to generate the chemical shift lists in order to minimize chemical shift deviation during subsequent NMR structural calculations. The BMRB accession numbers are 19047 and 19048 for BcII and the BcII–thiomandelate complex respectively.
